# Is Brazil prepared for the new era of infectious disease epidemics?

**DOI:** 10.1590/0037-8682-0218-2020

**Published:** 2020-04-30

**Authors:** Creuza Rachel Vicente

**Affiliations:** 1 Universidade Federal do Espírito Santo, Departamento de Medicina Social, Vitória, ES, Brasil.

Dear Editor:

The world has been facing a new era of emerging and re-emerging infectious diseases and the century XXI possibly will be marked by frequent, complex, and impactful epidemics^1^. Nowadays, an unprecedented pandemic related to severe acute respiratory syndrome coronavirus 2 (SARS-CoV-2) is imposing social and economic losses. Preventive measures, such as travel restrictions and quarantine for entire populations, are been adopted on a global scale, and the health systems of developing and developed countries are being overwhelmed due to the increase of services demand.

The health systems of all countries must be constantly prepared to deal with emerging and re-emerging infectious diseases, in an integrated cycle of preparation, response, and recovery^1^. Global health needs an efficient local response since infectious diseases present a fast spread in the globalized world. Therefore, the implementation of the International Health Regulations (IHR) by all nations, as well as the establishment of the Universal Health Coverage (UHC), are essential for dealing with this reality, as well as to achieve the Sustainable Developing Goals.

In Brazil, the Unified Health System (SUS) plays the main role in the preparation of the country for this new era. SUS has been acting in the response to the novel coronavirus disease 2019 (Covid-19) even before the identification of the first case in Latin America, with the declaration of national public health emergency^2^. Then, the Emergency Health Operations Centers for Covid-19 were activated to prepare organized and coordinated actions and advise the health sector on contingency plans and response measures to prevent the spread of the disease^2^.

In 2018, Brazil scored higher than the world average on IHR all capacities thanks to its universal public health system. Nevertheless, health service provision and points of entry were detected as its main challenges^3^. Zoonotic events and the human-animal interface, food safety, risk communication, national health emergency framework, and legislation and finance also may be improved to guarantee timely and effective prevention, detection, assessment, notification, reporting, and response to health risks and emergencies (Table 1).

**TABLE 1:** Scores of capacities related to International Health Regulations in 2018 and Universal Health Coverage in 2017, Brazil and World.

	Brazil	World
International Health Regulations*		
Legislation and financing	93%	62%
IHR coordination and national IHR focal point functions	100%	67%
Zoonotic events and the human-animal interface	80%	63%
Food safety	80%	61%
Laboratory	100%	70%
Surveillance	100%	71%
Human resources	100%	63%
National health emergency framework	87%	59%
Health service provision	47%	60%
Risk communication	80%	57%
Points of entry	60%	52%
Chemical events	100%	50%
Radiation emergencies	100%	52
All capacities average	87%	61%
**Universal Health Coverage** ^#^		
Index of Service Coverage^¶^	79%	66%
Infectious diseases control	70%	58%
Service capacity and access	99%	70%

Sources:*World Health Organization (2019) ^3^; **#**World Health Organization (2019)^4^. **¶**Index of service coverage considers reproductive, maternal, newborn and child health, infectious disease control, noncommunicable diseases, and service capacity and access^5^.

Brazil presented all indicators related to health service provision below the world average, with a little functional capacity. It reached a score of 40% for management of health emergency response operation (world = 57%) and capacity for infection prevention and control and chemical and radiation decontamination (world = 56%)^3^, indicating a compromised ability of response due to lack of case management and infection control^6^. The country also scored 60% for access to essential health services (world = 66%)^3^, which affects the ability to prevent, detect, and control infectious disease outbreaks^6^. The improvement of this capacity is essential to provide critical services to maintain local populations healthy and safe, not only for protecting against cross-border outbreaks^7^. The indicators related to UHC (Table 1) demonstrate that Brazil needs to improve infectious disease control, including basic sanitation, which depends on sustained intersectoral investments. In the Covid-19 response, availability of intensive care units and mechanical ventilators are concerns regarding health service provision^2^.

Core capacity requirements at all times for designated airports, ports and ground crossings, and effective public health response at points of entry, both indicators related to points of entry capacity, scored 60% in Brazil (world = 55% and 48%, respectively)^3^, indicating low effectiveness on prevention and control measures at the subnational level^6^. All points of entry must be provided with the necessary capacities to deal with travelers, animals, and cargo transported since they could play a role as reservoirs or vectors for different pathogens^8^. Many viruses circulating currently in Brazil, such as SARS-CoV-2, Zika, and Chikungunya, were imported from other countries in recent years^9^. Therefore, the National Agency of Sanitary Surveillance (ANVISA), the main responsible for the activities related to IHR at the Brazilian points of entry, must receive increasing investments to improve core capacities, enabling effective response.

Brazil scored 80% for the indicators collaborative effort on activities to address zoonosis (world = 63%), multisectoral collaboration mechanism for food safety events (world = 61%), and capacity for emergency risk communications (world = 57)^3^. Therefore, the country has the national and subnational functional capacity to deal with diverse health events, providing preventive measures, but needs improvement to be considered well advanced and sustainable in these areas^6^.

Zoonotic events and the human-animal interface are important capacities considering the emerging infectious diseases since 75% of the pathogens related to them have an animal origin^10^. The improvement of this capacity may permit to attain higher proportion zoonotic events, detecting animal reservoir, and vectors timely. Since food may be a vehicle for various pathogens, developing food safety capacity collaborates to prevent infection outbreaks^6^. The One Health approach must be emphasized in the country's health system to address these two points. Risk communication also must be improved to reach out to communities at the local, national, and global levels, encouraging their participation.

In Brazil, management of health emergency response operation scored 60% (world = 64%) and was the only of the three indicators related to the national health emergency framework with less than 100%^3^, indicating the necessity of improvement to incident management systems for public health events at the subnational level^6^. Besides, the country scored 80% for financing mechanisms and funds for the timely response to public health emergencies (world = 63%), one of the three indicators related to legislation and finance capacity^3^. This indicator is related to the availability of access to finance, which must be improved especially at the sub-national level^6^.

Despite the high score in the laboratory capacity, it has been a fragile point to respond to Covid-19, with Brazilian National Laboratory Network having the insufficient capability to perform the tests necessary for dealing with the incident cases^11^, particularly regarding RT-PCR^2^. Therefore, it raises the question of the overestimation of the real capacity by country self-assessment^7^.

Brazil must maintain a focus on enhancing the capacities related to IHR and UHC, especially those in deficit, and, at the same time, develop measures to prevent outbreaks related to emerging and re-emerging infectious diseases, targeting animals, human sentinels for spillover events, and the general human population^12^. Thus, the country will be more prepared for a globalized world marked by alterations in the environment and human behavior, urbanization, climate change, and increased travel, factors that contribute to the challenging infectious diseases epidemics.
